# Exploring the Human–Animal Interaction (HAI) for Children with ASD Across Countries: A Systematic Review

**DOI:** 10.1007/s10803-025-06745-8

**Published:** 2025-03-03

**Authors:** Hiu Wo Chan, Lucy Shih Ju Hsu, Kathy Kar Man Shum

**Affiliations:** Department of Psychology, The University of Hong Kong, Pok Fu Lam, Hong Kong

**Keywords:** Human–animal interaction, Companion animals, Therapy animals, Children with ASD

## Abstract

Human–Animal Interaction (HAI) has been widely adopted as an approach to enhance the well-being of children with ASD, who often experience significant social impairments, emotional dysregulation, and other daily challenges. Given the potential variation of HAI across countries, there is a particular need to explore this phenomenon within different cultural contexts and to illuminate directions for facilitating positive HAI among children with ASD. The purpose of this review is to systematically synthesize the current knowledge of HAI as applied to children with ASD and to discuss possible variations across different cultural contexts. A systematic database search was conducted to synthesize HAI characteristics from existing studies that met the selection criteria. The results highlighted that most of the selected studies (*N* = 97) were conducted in Europe or the United States. The most common format of HAI identified was animal-assisted intervention, followed by pet ownership. Dogs and horses were the primary animals involved in HAI for children with ASD, while the majority of HAI occurred in home settings and at horse riding or training centres. To conclude, this review provides a more comprehensive lens for understanding the phenomenon of HAI for children with ASD across different countries and discusses cultural variations in terms of the companion animals involved, the formats, and the settings of HAI. It also offers therapeutic insights into the multicultural aspects of HAI, which may shed light on future interventions for children with ASD through HAI in more diverse settings.

Autism Spectrum Disorder (ASD) is a heterogeneous condition characterized by significant developmental challenges in social interaction, verbal and nonverbal communication, and repetitive behaviors that are not explained by intellectual disorders or developmental delays, as defined by the Diagnostic and Statistical Manual of Mental Disorders, Fifth Edition, Text Revision (DSM-5-TR; American Psychological Association, 2024). Human–Animal Interaction (HAI) refers to the mutual bond, dynamic exchange, and relationship that develops between animals and humans (Esposito et al., 2011; Griffin et al., 2011). HAI has been widely introduced in different contexts, including homes, schools, clinical settings, laboratories, agriculture, and natural environments (Hosey & Melfi, 2014), providing various social and emotional benefits for children with ASD (Abd Manan, 2023; Becker et al., 2017).

As animals typically show their intentions through behaviors, HAI is often simpler than interactions with humans. Children with ASD tend to interpret animal behaviors based on their movements, offering them opportunities to learn how to engage in basic social communication (Byström et al., 2019). Significant improvements have been observed in the social skills of children with ASD, along with a decrease in social withdrawal behaviors, after therapeutic sessions with animals, such as guinea pigs (O'Haire et al., 2014). Moreover, interaction with animals can activate oxytocin, which has anti-stress effects and facilitates social interaction (Beetz et al., 2012). This is crucial for children with ASD as it helps to alleviate anxiety or other negative emotions they may experience during social interactions. When a companion animal is present during therapy sessions, children with ASD often display less distraction, a more playful mood, and better engagement with the therapist (Martin & Farnum, 2002).

Interestingly, the modality of HAI adopted may differ across countries depending on the cultural context. Specifically, to align with the suitable approaches and preferences of children with ASD, factors such as the format, types of companion animals used, and other aspects of HAI should be carefully considered. Although some previous studies have investigated the multicultural context of HAI, none have examined its cultural nuances for children with ASD across different countries. This underscores the importance of synthesizing existing studies for a comprehensive understanding of HAI for children with ASD, thereby identifying potential cultural variations for more tailored and effective localized practices.

## Overview of Human–Animal Interaction (HAI)

HAI has been observed and studied globally across decades and cultures (Fine, 2015). Given the potential benefits associated with HAI, companion animals have been widely incorporated into various contexts and practices. Different terminologies have emerged within the diverse subfields under the HAI umbrella. According to varying natures and goals, HAI can be classified into two categories: affiliative relationships and animal-assisted interventions (such as animal-assisted education, animal-assisted therapy, animal-facilitated activities, animal-assisted play therapy, etc.). Both formats may involve animals with or without training.

An affiliative relationship entails the establishment of a strong emotional bond with companion animals through pet ownership. In contrast, animal-assisted interventions (AAI) involve professional practitioners (e.g., therapists, handlers, teachers) facilitating interactions between animals and individuals in specific settings to achieve psychological, physiological, or educational benefits. Companion animals are believed to serve as therapeutic aides or facilitators in intervention settings to attain therapeutic goals (Martin & Farnum, 2002; Zilcha-Mano et al., 2011). Those companion animals trained to perform specific tasks during HAI for clinical, educational, or other therapeutic purposes are regarded as service animals. While the terms “pet” and “companion animal” are often used interchangeably (Bures et al., 2021b), professionals in veterinary medicine and animal welfare commonly refer to “companion animals” as those involved in a dyadic psychological bond and mutual relationship with humans (Walsh, 2009). Common companion animals found across various subfields of HAI include dogs, cats, guinea pigs, horses, hamsters, birds, etc.

## Benefits of HAI for Children with ASD

During childhood and preadolescence, companion animals are often considered crucial assets for nurturing developmental needs (Davis & Juhasz, 1985). Interacting with companion animals improves human well-being across social, emotional, cognitive, and physical dimensions (Droboniku & Mychailyszyn, 2021). Children with developmental disabilities, especially those with ASD, are frequently among the populations to which HAI is employed. O’Haire (2013) has identified positive social and emotional outcomes for children with ASD, including enhanced social interaction, communication, and stress reduction. The presence of companion animals, such as dogs, can serve as a catalyst for facilitating social engagement and creating a more relaxing environment, potentially easing anxiety and irritability (Berry et al., 2013; O’Haire, 2013). As children with ASD often face challenges in social and emotional domains, HAI is considered a promising approach to help alleviate associated autistic symptoms.

Despite these potential benefits, there remains a lack of comprehensive reviews examining various features of HAI for children with ASD, such as formats, settings, and types of companion animals involved, which may be culturally context-dependent. Hence, before introducing tailored HAI practices for children with ASD, it is essential to develop a thorough understanding of the multicultural considerations of HAI for this population in different countries. A holistic view of HAI practices worldwide, derived from existing research, can enhance current knowledge and promote more adaptive HAI practices for children with ASD across diverse cultural contexts.

## Societal and Cultural Influences on HAI

Culture refers to the set of collective values, patterns of attitudes, beliefs, and behaviors that characterize members of a particular societal group (Harris, 1993). These shared perceptions are commonly based on individuals’ race, nationality, and ethnicity (Godfrey et al., 2020). Cross-cultural differences in social norms and practices are believed to play a vital role in shaping how humans perceive the value of animals and how humans interact and build relations with them (Amiot & Bastian, 2015; Busch et al., 2022).

A study by Gray and Young (2011) revealed that pets were treated as “friends” and “family members” in only five out of 60 cultures, suggesting that the perception of household pets in intimate roles can vary across cultures. Social norms and practices may also determine the types of companion animals preferred for pet keeping in different countries (Herzog & Foster, 2010). For example, dogs are typically kept as household pets in the US, whereas in some Asian countries, dog meat consumption is not entirely prohibited, illustrating contrasting cultural attitudes toward dogs (Pettier, 2021). Similarly, stag beetles are considered pets in Japan but do not hold the same status in the US (Arluke & Sanders, 1996).

Moreover, cultural norms regarding social engagement and emotional expression can significantly impact how children connect socially and emotionally with companion animals. For example, East Asian cultures emphasize the importance of interpersonal space during interactions, while Western cultures may perceive this as a lack of social engagement (Golson et al., 2022). In cultures with high individualism, such as the US, people tend to be more emotionally expressive when interacting with others, in contrast to collectivistic cultures in some Asian countries (Matsumoto et al., 2008). Through social modeling, children with ASD may acquire culture-specific bonding skills that could potentially be generalized from human interactions to interactions with animals. Hence, children with ASD in various countries may adopt different approaches to socially and emotionally connect with companion animals in the context of HAI.

In AAI, incorporating therapy animals into the intervention process can help facilitate the therapeutic alliance and create a calmer therapeutic atmosphere for children with ASD. However, cultural differences in attitudes toward animals may influence the therapeutic relationship and the acceptance of interventions related to HAI (Sheade & Chandler, 2014). Specifically, cultural factors may affect children’s willingness to work with therapy animals, which can enhance or impede the building of triadic relationship between therapists, animals, and children during AAI (Chandler, 2012). Some children may feel uncomfortable interacting with certain types of companion animals. For instance, in Beijing, some large dog breeds over 35 cm in height are not permitted as pets or allowed in public places. Consequently, children from societies where interactions with large animals are limited may feel anxious when engaging with a large therapy dog. This discomfort can hinder their engagement and trust-building during therapy sessions. Given that children with ASD already face more challenges in connecting with their therapists compared to typically developing children (Klebanoff et al., 2019), societal norms can further complicate the implementation of AAI for children with ASD.

In addition, the interpretation of autistic symptoms can vary across countries, leading to differing views on typical behaviors. For example, in certain African or Latino cultures, avoiding direct eye contact with figures of authority is seen as a sign of respect rather than an autistic symptom (de Leeuw et al., 2020). Hence, the goal of facilitating eye contact through HAI, such as involving therapy dogs in social initiation tasks, may not be a priority for children with ASD in those cultures. Therefore, it is crucial for AAI practitioners in various countries to be mindful of these cultural nuances and tailor interventions accordingly to effectively address the unique needs of specific cultural groups (Sheade & Chandler, 2014).

## Knowledge Gap

As culture can significantly influence the multifaceted nature of HAI, it is important to explore the phenomenon of HAI under different cultural contexts to inform more adaptive HAI practices for children with ASD. Although HAI has been widely practiced among various target groups worldwide, the concept of HAI remains relatively novel in Asia compared to Western countries. Particularly, it is believed that the awareness of emotions and attachment to animals in Chinese culture is less common than in Western societies (Su & Martens, 2020). This less favorable attitude towards companion animals also hampers the facilitation of HAI in Chinese culture. While Japan is considered to have a better-established development of HAI, related studies are primarily published in non-English journals, which may hinder the knowledge-building of HAI in the Asian context. Hence, refined knowledge concerning the practice of HAI across different countries may serve as a valuable reference for developing a more holistic framework of HAI in Asia.

Furthermore, while most previous reviews emphasize examining the multicultural variation of HAI, none specifically focus on its practice within the autistic population. The social and emotional support needs of children with ASD may differ from those of neurotypical children, suggesting that the involvement of companion animals, as well as the formats and settings concerning HAI, could vary. For example, autism assistance dogs are specifically trained to provide daily support for families with children who have ASD and to enhance their social competence. Given this cultural diversity, a thorough understanding of the potential cultural variations in HAI across countries is necessary to achieve the “goodness of fit” required to address the needs of children with ASD. Since social and emotional needs are significant concerns for these children, the benefits of HAI in these two domains are, therefore, the scope of focus in this review.

## Objectives

In light of the aforementioned knowledge gap, this study aims to (1) systematically synthesize current knowledge on HAI in the context of children with ASD across various countries; (2) explore potential cultural variations in HAI practices among different countries, with the goal of identifying insights for better applications of HAI for children with ASD. The key research questions to be addressed include: (1) Which companion animals and formats of HAI are highlighted in existing studies on children with ASD across different countries? (2) What cultural factors might contribute to variations in HAI practices for children with ASD across countries?

## Method

This systematic review used the Preferred Reporting Items for Systematic Reviews and Meta-Analyses (PRISMA) guidelines (Page et al., 2021) to inform the protocol for the search strategy, inclusion and exclusion criteria, and data extraction.

### Search Strategy

Studies were identified and searched in various electronic databases, including PubMed, Scopus, ProQuest, EBSCOhost, and Web of Science. The publication period of the included papers was set from 1 January 2000 to 31 December 2023. The key search terms for these databases included a combination of at least one identifier for HAI, one identifier for animal-related terms, one identifier for children with ASD, and one identifier for social and emotional outcomes (Table 1). The designs of the identified studies could be quantitative, qualitative, or mixed methods.

**Table 1:** Key search terms of identifiers

Identifier for HAI	“ownership” OR “therapy” OR “companion” OR “assist” OR “service” OR “therapeutic”
Identifier for animal-related terms	“animal” OR “pet” OR “dog” OR “canine” OR “cat” OR “equine” OR “horse” OR “horseback” OR “dolphin”
Identifier for study target	“child” AND “ASD” OR “autism” OR “autistic” OR “asperger”
Identifier for social and emotional outcomes	“social” OR “emotion” OR “communication”

### Inclusion and Exclusion Criteria

The title and the abstract were initially screened for eligibility for inclusion by the first author. Studies with titles and abstracts meeting the inclusion criteria were obtained, while some required a more thorough review of the full text to examine their eligibility for inclusion. The inclusion criteria used for the review selection were as follows: (1) involved empirical data; (2) involved any type of HAI; (3) targeted children under 18 years old with a diagnosis of ASD; and (4) measured social and/or emotional outcomes.

After the initial screening, studies were further reviewed based on the full text. Studies were excluded if they: (1) were review papers or meta-analyses; (2) were not peer-reviewed; (3) were not published in English; (4) lacked a detailed methodology; (5) were not relevant to HAI; or (6) involved adult participants or children without ASD, but separate data were not generated.

### Data Extraction and Analysis

Key data were identified and extracted from each study based on the objectives of this review. To examine the study characteristics, the extracted items included the country of origin, HAI terminology, type of companion animal involved, HAI format, and HAI setting. Given the cultural diversity across nations, the data were primarily summarized through country-based analysis to obtain a refined understanding of the potential cultural variations in HAI.

## Results

### Description of Studies

**Fig. 1:**
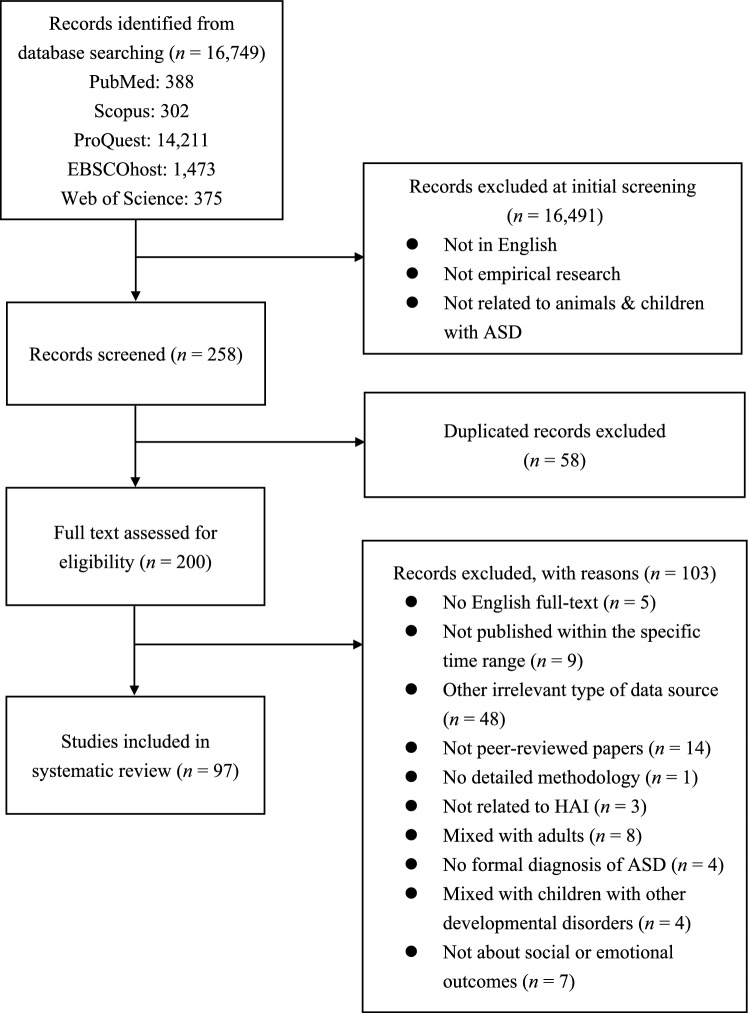
Flow diagram of study selection process

Figure 1 shows the flow diagram of the study selection process. The key features of HAI in the selected studies were extracted and summarized in Table 2. Sixty out of the 97 articles were published in the 2010s, while 33 were published between 2020 and 2023. Only four were published between 2000 and 2009. This may indicate a growing interest in publications on the topic of HAI for children and adolescents with ASD in recent years.

**Table 2:** Overview of the included studies

Study	Authors	Country	HAI terminology	Type of Companion animal	Format of HAI	Setting of HAI	Study Design	Children’s Gender & Age	N (treatment/ control)	Social and (or) emotional outcomes
1	Abihsira et al. (2020)	Canada	Therapeutic horseback riding	Horse	AAI (with untrained animal)	Horse riding facilities	Quantitative study	M = 11, F = 11 Mean age = 10.5 (Aged 6–18)	22	Significant improvements in social skill domains, including receptive language, self-regulation domains and pro-social behaviors (after controlling for severity level of autism and previous therapeutic horseback riding experience)
2	Adkins et al. (2023)	USA	Ownership of a pet dog	Dog	Pet ownership (with untrained animal)	Home	Qualitative study	M = 8, F = 1; other gender = 1 Mean age = 10.7 (Median age = 9; Aged 8–17)	10	Strong bond and positive relationship were found between the child and the dog due to the love as well as the protective, attentive and gentle nature of their dogs. Dogs’ attention, companionship and bonding time helped children with ASD meet the challenges resulting from autism Child’s enjoyment in caring for their dog and successful integration of dogs within family routines were found as the positive outcomes for families with children with ASD
3	Ajzenman et al. (2013)	USA	Hippotherapy	Horse	AAI (with untrained animal)	Horse arena	Quantitative study	M = 4, F = 3 Mean age = 8.4 (*SD* = 2.5)	7	Increase in the overall adaptive behaviors in receptive communication and coping, social leisure
4	Al-Hmouz and Arabiat (2015)	Mutah (Jordan)	Therapeutic horseback riding	Horse	AAI (with untrained animal)	Horseback riding training centre	Quantitative study, Participants randomly assigned to the experiment & control group	Treatment: M = 16, F = 7; Mean age = 11.75 (*SD* = 1.08) Control: M = 14, F = 8; Mean age = 11.91 (*SD* = 1.38)	45 (23/22)	Reduction in stereotyped behaviors, improvement in communication and social interaction, drop in autistic behaviors
5	Anderson and Meints (2016)	UK	Equine-assisted activity	Horse	AAI (with untrained animal)	Horse centre	Quantitative study	M = 11, F = 4 Mean age = 10 (*SD* = 3.8)	15	Improvement in social functioning, maladaptive behavior traits and empathy
6	Appleby et al. (2022)	Australia	Ownership of an autism assistance dog	Dog	Pet ownership (with trained animal)	Home	Qualitative descriptive approach with the use of occupation mapping	Gender not mentioned Aged 7–12	8	Facilitated the development of empathy and social skills, building of confidence Increased calmness, decreased stress-related behaviors, and decrease in the number, severity and length of meltdowns
7	Ávila-Álvarez et al. (2020)	Spain	Animal-assisted intervention	Dog	AAI (with trained animal)	Child rehabilitation and early care unit at hospital	Quantitative study	M = 13, F = 6 Mean age = 46.2 months (*SD* = 12.6)	19	Significant improvement in communication & social interaction skills, child-dog social relationship and child-therapist relationships
8	Bass et al. (2009)	USA	Therapeutic horseback riding	Horse	AAI (with untrained animal)	Equestrian training centre	Quantitative study, with waitlist-control group	Treatment: M = 17, F = 2; Mean age = 6.95 (*SD* = 1.67) Control: M = 12, F = 3; Mean age = 7.73 (*SD* = 1.65)	34 (19/15)	Exhibited greater sensory seeking, sensory sensitivity, social motivation, less inattention, distractibility and sedentary behaviors
9	Becker et al. (2017)	USA	Animal-assisted social skills training	Dog	AAI (with trained animal)	School setting	Quantitative study, with control group (traditional social skills group)	M = 28, F = 3 Mean age = 10.97 (*SD* = 1.84)	31(17/14)	Exhibited fewer social skill deficits, fewer restricted and repetitive behaviors and more typical social communication, better social responsiveness (playing appropriately with others, making eye contact, initiating interactions with peers) Significant reduction in depressive symptoms (negative mood/physical problems, negative self-esteem, ineffectiveness and interpersonal problems)
10	Ben-Itzchak and Zachor (2021)	Israel	Dog training intervention	Dog	AAI (with trained animal)	School setting (special education school)	Quantitative study with waitlist control group	Treatment: M = 29, F = 8; Mean age = 5:4 years (SD = 0:10) Control: M = 32, F = 4; Mean age = 4:4 (SD = 0:11)	73 (37/36)	Improvement in adaptive social and communication skills Partial improvement in the anxiety symptoms
11	Borgi et al. (2016)	Italy	Equine-assisted activity	Horse	AAI (with trained animal)	Equestrian riding centre	Quantitative study with waitlist control group	Treatment: M = 15; Mean age = 9.2 years (SD = 1.8) Control: M = 13; Mean age = 8.0 (SD = 1.5)	28 (15/13)	Improvement in social functioning, ameliorated executive abilities, reduced latency of the first move during a problem-solving task
12	Burrows et al. (2008)	Canada	Ownership of a service dog	Dog	Pet ownership (with trained animal)	Home	Qualitative study	M = 7, F = 3 Aged 4.5–14	10	Facilitated appropriate affection and interactions with others Decreased anxiety, increased calmness, reduced in the number of meltdowns or tantrums, dissipated anger and more manageable bedtime routines, increased happiness
13	Byström et al. (2019)	Sweden	Nature- and animal-based interaction and communication treatment	Non-specific (horse, dog, cat, bird)	AAI (with untrained animal)	Farm	Qualitative study	Unknown gender Aged 6–8	9	Reduced stress and provided calmness, increased ability to reflect in conversation, engage socially and use more fantasy in play, improved capacity to engage in a more affective based communication style Displayed much joy and laughter by watching the animals
14	Carlisle (2014)	USA	Pet ownership	Dog	Pet ownership (untrained animal)	Home	Qualitative study	Mean age = 13	70	47% of parents mentioned companionship as a benefit of owning dogs and one parent emphasized this with stress relief
15	Carlisle (2015)	USA	Ownership of a pet dog	Dog	Pet ownership (with untrained animal)	Home	Quantitative study with control group (no dog)	M = 65, F = 5 Mean age = 13	70 (23/47)	Significant increase in social skills for the subscale item of assertion
16	Carlisle et al. (2018)	USA	Pet ownership	Non-specific (dog, cat)	Pet ownership (with untrained animal)	Home	Qualitative study	M = 31, F = 306; unknown gender = 1 Mean age = 43	338	Increased sibling interaction and more family time, provided learning opportunities (e.g., social and communication skills, empathy and responsibility), relief in stress or agitation
17	Carlisle et al. (2020)	USA	Pet ownership	Non-specific (dog, cat)	Pet ownership (with untrained animal)	Home	Qualitative study	M = 598, F = 166 Mean age = 12.52 (*SD* = 3.9)	764	Strong bond building, with 71.1% of children being attached to their companion animals Increased happiness with great companion
18	Carlisle et al. (2021)	USA	Ownership of a cat	Cat	Pet ownership (with untrained animal)	Home	Quantitative study with control group (delayed to adopt a cat)	Treatment: M = 8, F = 2; Mean age = 9.00 Control: M = 5, F = 2; Mean age = 8.57	17 (10/7)	Greater empathy, significant increase in empathy and social skills Less separation anxiety
19	Carlisle et al. (2023)	USA	Ownership of a cat	Cat	Pet ownership (with untrained animal)	Home	Qualitative study	M = 8, F = 2 Mean age = 9.0	10	Improved social skills (e.g., social communication and cues, eye contact, responsibility) Enhanced companionship with laughter to each family member, improved mood when feeling upset, increased calmness with stress relief
20	Cerino et al. (2016)	Italy	Equine-assisted intervention	Horse	AAI (with untrained animal)	Horse riding centre and outdoor arena	Qualitative study (case study)	M = 1 Age = 8	1	Reduced the avoidance of contact with the present and to hide in imaginative past and future
21	Collacchi et al. (2023)	Italy	Equine-assisted activity	Horse	AAI (with trained animal)	Certified riding centre	Quantitative study with control group (typically developing children)	Treatment: M = 15, F = 4 Control: M = 11, F = 8 Aged 5–17	38 (19/19)	Elicited social interaction and communicative behaviors independent of gender and age
22	Coman et al. (2018)	USA	Equine-assisted activity	Horse	AAI (with untrained animal)	Equestrian training centre	Quantitative study with waitlist control group	Treatment: M = 19, F = 6; Mean age = 8.84 (*SD* = 1.72) Control: M = 23, F = 2; Mean age = 8.56 (*SD* = 1.5)	50 (25/25)	Improvement in overall social functioning, including social cognition, social communication, social motivation, and autistic mannerisms
23	Dollion et al. (2021)	Canada	Interaction with a service dog	Dog	AAI (with trained animal)	Room at a foundation	Quantitative study	Study 1: M = 14, F = 2; Mean age = 8.5 (*SD* = 0.7) Study 2: M = 3, F = 3; Mean age = 9.3 (*SD* = 1.1)	Study 1:16 Study 2: 6	First study: Initiated more frequently by the dog, more physical contact with the dog, spoke and vocalized more to the dog compared to the parents Second study: Spent more time gazing at the service dog, performed fixation more frequently on the dog than other objects
24	Dollion et al. (2022)	Canada	Ownership of a service dog	Dog	Pet ownership (with trained animal)	Home	Quantitative study with control group (without a service dog)	Treatment: M = 14, F = 1; Mean age = 166.7 months (*SD* = 28.2 months) Control: M = 9, F = 6; Mean age = 141.4 months (*SD* = 33.7 months)	30 (15/15)	Children with a service dog recognized joy more efficiently (i.e. higher accuracy and shorter reaction times) compared to negative expressions, notably fear and sadness than children without a service dog Children with a service dog were slightly quicker to recognize anger and displayed more suited and more differentiated visual scanning strategies when processing facial expressions
25	Figueiredo et al. (2023)	Brazil	Canine-assisted occupational therapy	Dog	AAI (with trained animal)	Outdoor sports court	Quantitative and qualitative studies	M = 1 Age = 6	1	Increase in the intrinsic motivation and frequency of showing attention, interest, persistence, communication, proximity and time close to the dog
26	Funahashi et al. (2014)	Japan	Animal-assisted activity	Dog	AAI (with trained animal)	Experimental room	Quantitative study with control group (typically developed children)	Treatment: M = 1 Control: M = 1 Age = 10	2 (1/1)	Increased positive social behaviors and decreased negative social behaviors
27	Fung (2014)	Hong Kong	Animal-assisted play therapy	Dog	AAI (with trained animal)	Multi-purpose room at school	Quantitative study	M = 8, F = 2 Mean age = 8.9 (Aged 7–10)	10	Children’s social behaviors were similar in the both treatment and control groups, but with a tendency of more positive verbal behaviors in the treatment group Children in the treatment group showed significantly less negative behavior toward the therapy dog compared with the behavior toward the doll on the comparison group
28	Fung and Leung (2014)	Hong Kong	Animal-assisted play therapy	Dog	AAI (with trained animal)	Multipurpose room at school	Quantitative study	Treatment: M = 4, F = 1; Mean age = 9.0 Control: M = 4, F = 1; Mean age = 8.8	10	Small but significant increase in the verbal social behavior, with positive change in questioning, verbal response, needing expression and sharing
29	Fung (2015)	Hong Kong	Animal-assisted play therapy	Dog	AAI (with trained animal)	Multipurpose room at school	Quantitative study	M = 1 Age = 7	1	Increased social communication, more joint-attention and waiting behaviors, lower rate of isolative behaviors
30	Gabriels et al. (2012)	USA	Therapeutic horseback riding	Horse	AAI (with untrained animal)	Horse riding centre	Quantitative study with wait-list control	Treatment: M = 21, F = 5; Mean age = 8.6 Control: M = 15, F = 1; Mean age = 8.8	42 (26/16)	Significant improvements in children’s irritability, lethargy, stereotypic behavior, hyperactivity, expressive language skills, motor skills, and verbal praxis/motor planning skills after the sessions Significant improvements in self-regulation behaviors in treatment group comparing with the waitlist control group
31	Gabriels et al. (2015)	USA	Therapeutic horseback riding	Horse	AAI (with untrained animal)	Horse riding centre	Quantitative study with control group (barn activity without horses)	Treatment: M = 49, F = 9; Mean age = 10.5 (*SD* = 3.2) Control: M = 52, F = 6; Mean age = 10.0 (*SD* = 2.7)	116 (58/58)	Increased social cognition, social communication along with the total number of words and new words spoken during a standardized language sample
32	Gabriels et al. (2018)	USA	Therapeutic horseback riding	Horse	AAI (with untrained animal)	Horse riding centre	Quantitative study with control group (barn activity without horses)	Treatment: M = 7, F = 29; Mean age = 10.7 (*SD* = 2.9) Control: M = 3, F = 25; Mean age = 9.4 (*SD* = 2.5)	64 (36/28)	Significant improvement in irritability and hyperactivity behaviors, social responsiveness (social cognition & communication), used a greater number of different words
33	García-Gómez et al. (2014)	Spain	Adapted therapeutic horse-riding	Horse	AAI (with untrained animal)	Equestrian centre	Quantitative study with “quasi-control” group	M = 13, F = 3 Aged 7–14	16 (8/8)	Significant improvement in the domains of aggressiveness, interpersonal relations and social inclusion in the experimental group compared to the control group
34	Germone et al. (2019)	USA	Animal-assisted activity	Dog	AAI (with trained animal)	Classroom in a hospital unit	Quantitative study with control group (a novel toy and toy handler)	M = 53, F = 13; unknown gender = 1 Mean age = 11.7 (*SD* = 3.5)	67	Demonstrated more social communication, more overall communication directed towards adult dog handler Displayed more positive statement, more gestures towards adult handler, more socially directed eye gazes towards peers and adult handlers More direct verbalizations to the dogs than to the toys Demonstrated more emotional displays (e.g., facial expressions, more smiling, laughing behaviors)
35	Ghorban et al. (2013)	Iran	Therapeutic horseback riding	Horse	AAI (with untrained animal)	Area of horseback riding	Quantitative study	M = 1, F = 5 Mean age = 8.5 (*SD* = 2.35)	6	Significant improvement in social skills, affective understanding/perspective taking, initiating and maintaining interaction
36	Grandgeorge et al., (2012a)	France	Interaction with a guinea pig	Guinea pig	AAI (with untrained animal)	Experimental setting at home	Quantitative study with control group (typically developed children)	Treatment: M = 27; Mean age = 9.6 (*SD* = 1.8) Control: M = 27, F = 32; Mean age = 9.4 (*SD* = 2.1)	86 (27/59)	More children with autism smiled at the animal and talked to parents/ observers about the animal or other topics
37	Grandgeorge et al., (2012b)	France	Pet ownership	Non-specific (dog, cat and/or little furry animal)	Pet ownership (with untrained animal)	Home	Quantitative study with control group (non-pet owners)	Treatment: M = 13; F = 7 Control: M = 13, F = 7; Aged 73 months to 201 months	40 (20/20)	Improvement in prosocial behaviors of individuals with autism
38	Grandgeorge et al. (2015)	France	Interaction with guinea pigs	Guinea pig	AAI (with untrained animal)	Home	Quantitative study with control group (typically developed children)	Treatment: M = 30, F = 1; Mean age = 9.5 (*SD* = 1.8) Control: M = 27, F = 32; Mean age = 9.4 (*SD* = 2.1)	90 (31/59)	More attracted to the pet if having privileged relationship with animals in previous experience
39	Grandgeorge et al. (2017)	France	AAI	Dog	AAI (with trained animal)	Not mentioned	Study 1: quantitative study with control group (attention shift of the animal trainer from the dog-child to the dog only) vs (attention maintained on the dyad) Study 2: quantitative study (within-subjects repeated measure)	Study 1: Treatment (M = 9, F = 1);Control (M = 9, F = 1); Mean age = 7.6 years (SD = 1.6) Study 2: M = 8, F = 1; Mean age = 13.7 years (SD = 2.3)	Study 1: 20 (10/10) Study 2: 9	Study 1: Increased overall visual attention of the ASD children when the animal trainer concentrating on the dog More sensitivity to the focus of visual attention of a human trainer and service dog as observed when the attentional focus shifted, and more orientation towards the animal trainer and the dog, contrary to the control group Study 2: Displayed more visual attention (duration of gazes) towards the service dog-animal trainer dyad in a social rivalry situation
40	Grandgeorge et al. (2019)	France	Interaction with guinea pigs	Guinea pig	AAI (with untrained animal)	Experimental room with equipment and standard cage setting	Quantitative study with control group (typically developed children)	Treatment: M = 14, F = 8; Mean age = 9.4 (*SD* = 0.4) Control: M = 22; Mean age = 9.3 (*SD* = 0.4)	44 (22/22)	Higher variability in the number of social initiations by guinea pigs Attempted to touch the guinea pigs more frequently
41	Grandgeorge et al. (2020)	France	Pet ownership	Non-specific (dog, cat)	Pet ownership (with untrained animal)	Home	Quantitative study with control group (typically developed children)	Treatment: F = 22; Dog owners’ mean age = 10.1 (*SD* = 2.1) Cat owners’ mean age = 7.5 (*SD* = 2.2) Control: M = 11, F = 9; Dog owners’ mean age = 9.4 (*SD* = 2.4) Cat owners’ mean age = 9.0 (*SD* = 1.9)	42 (22/20)	More gazes than glances towards the animals, whatever the species Displayed much more visual attention with pet cats than with pet dogs and the same amount of visual attention towards their pets, whatever the species
42	Griffioen et al. (2019)	The Netherlands	Dolphin-assisted therapy	Dolphin	AAI (with untrained animal)	Dolphinarium	Quantitative study	M = 4, F = 1 Mean age = 7.5	5	Increase in reasonable verbal communication skills and synchrony (adequate turn-taking) in all dyads, given that not all children improved equally
43	Griffioen et al. (2020)	The Netherlands	Dog-assisted therapy	Dog	AAI (with trained animal)	Therapy room	Quantitative study with control group (children with down syndrome)	Treatment: M = 4, F = 1; Mean age = 12 Control: M = 4, F = 1; Mean age = 14	10 (5/5)	Significant increase in synchrony between children and the therapy dog over time Increase in the emotional and behavioral problems
44	Grigore and Bazgan (2017)	Romania	Animal-assisted therapy	Dog	AAI (with trained animal)	Therapeutic centre	Qualitative study	M = 3, F = 1 Mean age = 6	4	Communicated more often and easily with adults, more responsive and visual contact Increased frequency of comply with simple rule, reduced frequency of disturbing others Reduced psychomotor agitation, increased expression of feelings and increased change of facial expression More appropriate emotional reactions, more emotional association with facial expressions and words, reduced refusal of hugs More emotion recognition of their own emotions as well as others
45	Grigore and Rusu (2014)	Romania	Animal-assisted therapy	Dog	AAI (with trained animal)	Carpeted therapy room	Quantitative study	M = 2, F = 1 Mean age = 7.3 (Aged 7–8)	3	Increase in the frequency of social imitations in the presence of the therapy dog Decrease in the level of social prompt needed to perform appropriate social interactions
46	Guay et al. (2022)	Canada	Ownership of an autism assistance dog	Dog	Pet ownership (with trained animal)	Home	Quantitative and qualitative studies	M = 1 Age = 4:9	1	Increased ability to maintain social interaction Increased ability to gesturally and physically maintain as social interaction across time with the presence of the assistance dog Diminished frequency of withdrawal and isolation behaviors Slightly increased frequency of parallel play Improved communication skills and social interactions, more eye contact established with others, a longer play time with siblings and toy sharing in the presence of the dog
47	Guay et al. (2023)	Canada	Ownership of an assistance or companion dog	Dog	Pet ownership (with trained animal)	Home	Quantitative and qualitative studies	M = 64, F = 21 Mean age = 10.73 (*SD* = 3.67)	85	Developed a sense of self-confidence, responsibility, routine and autonomy Increased social communication and interaction, learned new social skills to analyze the dog’s reactions, increased willingness of outdoor activities, vacation and family activities Better management of anger and anxiety, decreased the frequency and intensity of emotional meltdowns, improved emotional regulation
48	Harris and Williams (2017)	UK	Horse riding intervention	Horse	AAI (with untrained animal)	Horse riding arena	Quantitative study with control group (with no horse riding session)	M = 22, F = 4 Treatment: Mean age = 8.2 (*SD* = 10.56) Control: Mean age = 7 (*SD* = 3.95)	26 (12/14)	Significant reduction in the severity of ASD symptoms and hyperactivity, greater change in social functioning, displayed positive level of engagement in the interaction with animals
49	Harwood et al. (2019)	Australia	Ownership of a dog	Dog	Pet ownership (with untrained animal)	Home	Qualitative study	M = 7, F = 6 Age 5–12	13	Allowed the connection with the social world and developed an appreciation of the life cycle Assisted the development of social skills (including empathy) Provided a calming influence on children, particularly when being distressed Building of love, companionship and positive relationship between the companion canine and children
50	Hellings et al. (2022)	Australia	Ownership of an assistance dog	Dog	Pet ownership (with trained animal)	Home	Qualitative study	M = 4 Aged 5–13	4	Increased socialization as well as participation in daily routines, family outings and activities The assistance dog provided the child with a sense of confidence and stayed aside throughout the outing, which helps regulate emotions and calm them when feeling upset
51	Hernández-Espeso et al. (2021)	Spain	Dolphin-assisted therapy	Dolphin	AAI (with untrained animal)	Dolphinarium	Quantitative study with control group (with no interaction with dolphins to support activities)	Treatment: M = 19, F = 5; Mean age = 52.9 Control: M = 14, F = 5; Mean age = 53.9	48 (24/19)	Significant improvement in communication skills, especially in the “frequency of vocalizations directed towards others” and “gestures” Improvement in comprehension skills, expression skills, communication skills and social skills
52	Hill et al. (2020)	Australia	Canine‐assisted occupational therapy	Dog	AAI (with trained animal)	Clinical setting	Qualitative study with waitlist control group	M = 7, F = 3 Age 4–6:11	10	Increased sense of competence when trying new things, displayed in social behaviors Increased motivation to participate within the therapy session and a willingness and excitement to engage Motivation in participating in nurturing behaviors with the therapy dog Provided a sense of acceptance for children with ASD
53	Holm et al. (2014)	USA	Therapeutic horseback riding	Horse	AAI (with untrained animal)	Therapeutic riding centre	Quantitative study	M = 3 Age 6–8	3	Increased verbal communication and verbalization at home and in the community
54	Jorgenson et al. (2020)	USA	Animal-assisted intervention	Dog	AAI (with trained animal)	Outpatient clinic	Quantitative study	M = 4, F = 1 Aged 3–8	5	For two participants, the contingent access to the therapy dog increased their social interactions. For one participant, the noncontingent access to the therapy dog slightly increased the verbal statements
55	Kalmbach et al. (2020)	USA	Occupational therapy in an equine environment	Horse	AAI (with untrained animal)	Therapy room, tack room and outdoor sensory trail	Qualitative study	M = 4 Mean age = 10 (Aged 8–13)	4	Increased occupational performance, social skills and frequencies of social interactions Increased calmness, fewer negative emotional episodes and chaos and handled everyday situations more smoothly
56	Kern et al. (2011)	USA	Equine-assisted activity	Horse	AAI (with untrained animal)	Equestrian training centre	Quantitative study	M = 18, F = 6 Mean age = 7.8 (*SD* = 2.9)	24	Marginal improvement in the parent–child interaction in terms of reduction of Negative Regard, and Mood & Tone
57	Kręgiel et al. (2019)	Poland	Animal-assisted therapy	Non-specific (cat, dolphin, horse, dog)	AAI (with untrained animal)	Not mentioned	Quantitative study	M = 12, F = 38 Mean age = 7.2 (*SD* = 4.5)	50	More animated gestures, and increased frequency of verbal reactions Positive effects on children’s emotion-related functioning, with an increased frequency of expression of emotions and feelings
58	Krskova et al. (2010)	Slovak Republic	Animal-asssisted therapy	Guinea pig	AAI (with untrained animal)	Special class at a primary school	Quantitative study with control group (with no therapeutic animal)	M = 5, F = 4 Mean age = 9.3 (Aged 6–13)	9	Significant increase in the frequency of contacts of autistic children with their acquaintances and unfamiliar person (observer) in the presence of guinea pig
59	Lanning et al. (2014)	USA	Equine-assisted activity	Horse	AAI (with untrained animal)	Not mentioned	Quantitative study with control group (social circles program)	Treatment: M = 9, F = 4; Mean age = 7.5 (*SD* = 3.2) Control: M = 12; Mean age = 9.8 (*SD* = 3.2)	25(13/12)	Positive treatment effects in areas of social functioning, physical functioning, school functioning, overall mental health and behavior Significant improvement in emotional function and quality of life domains
60	Leighton et al. (2023)	USA	Placement of a service dog	Dog	Pet ownership (with trained animal)	Home	Qualitative study with control group (without service dog)	Aged 5–17	50 (38/12)	Greater social inclusion for children and their families, and decreased experiences of judgement and stigma Perceived as family members, service dogs may co-regulate with the autistic child and family members, and can be a source of joyful connection within the family
61	Lisk et al. (2023)	USA	Pet ownership	Non-specific (dog, cat)	Pet ownership (with untrained animal)	Home	Qualitative study	M = 8, F = 2 Aged 4–17	10	Attained social growth through play with pets, particularly in areas of learning empathy and responsibility Gained a sense of responsibility from pet ownership, taking care of the pet Developed social skills of children with ASD particularly in how they interact with animals
62	Llambias et al. (2016)	Canada	Equine-assisted occupational therapy	Horse	AAI (with untrained animal)	Equestrian training centre	Quantitative study	M = 4, F = 3 Aged 4–8	7	Talked more, with more initiation of communication, new words, or longer sentences Significant improvement in engagement Showed signs of enjoyment such as smiles, laughing, or even singing while trotting
63	London et al. (2020)	Australia	Animal-assisted therapy	Dog	AAI (with trained animal)	Not mentioned	Qualitative study	M = 16, F = 1 Aged 4–19	17	Facilitated interactions and communications with others, decreased reticence around the dog, easier to communicate with the therapist, more community participation for children Consistent level of enjoyment during the sessions, removing the stress and pressure existed in interpersonal relationships
64	Malcolm et al. (2018)	UK	Equine therapy	Horse	AAI (with untrained animal)	Horse therapy centre	Qualitative study	Not mentioned	Not mentioned	Improved autistic symptoms Showed more empathy, social interaction with other people, more communicative and increasingly aware of self and others, with more eye contacts Reduced salience of issues around social interaction, communicative ability, and stereotypical behaviors
65	MdYusof and Chia (2012)	Singapore	Dolphin-assisted therapy	Dolphin	AAI (with trained animal)	Classroom and dolphin lagoon	Quantitative study (quasi-experimental study)	M = 10, F = 5 Aged 9–10	15	Extremely significant improvement in the reduction of stereotyped behaviors and autism quotients, improvement of communication and social interaction after the program
66	Michelotto et al. (2019)	Brazil	Animal-assisted activity	Dog	AAI (with trained animal)	Specialized therapeutic centre	Quantitative study	M = 14, F = 1 Mean age = 5.6 (*SD* = 1.6)	15	Positive modification in speech communication and creating & reduction in rituals, increased positive gestures and facial expressions
67	Morgan and O'Byrne (2023)	Ireland	Ownership of a certified canine	Dog	Pet ownership (with trained animal)	Home	Qualitative study	Not mentioned	9	An autism assistance canine could bring positive influence on the behavior, safety, social interaction, independent functioning, companionship, language development, educational experience and the family life for an autistic children
68	O'Haire et al., (2013a, 2013b)	Australia	Interaction with guinea pigs	Guinea pig	AAI (with untrained animal)	Classroom	Quantitative study with control group (presence of toys)	Treatment: M = 24, F = 9; Mean age = 9.4 (*SD* = 2.3) Control: M = 28, F = 38; Mean age = 9 (*SD* = 2.3)	99 (33/66)	Demonstrated more social approach behaviors (including talking, looking at faces, and making tactile contact) and received more social approaches from their peers, displayed more prosocial behaviors in the presence of animals Displayed more positive affect (smiling and laughing) and negative affect (frowning, crying, and whining), talked more about positive things (and less about negative things) in the presence of animals
69	O'Haire et al. (2014)	Australia	Animal-assisted activity	Guinea pig	AAI (with untrained animal)	Classroom at school	Quantitative study, with waitlist-control group	Treatment: M = 22, F = 5; Mean age = 8.2 (*SD* = 1.7) Control: M = 28, F = 9; Mean age = 9.5 (*SD* = 2.4)	64 (27/37)	Significant improvements in social functioning (e.g., increase in social approach behaviors and social skills, decrease in social withdrawal behaviors)
70	O'Haire et al. (2015)	Australia	Free play with guinea pigs	Guinea pig	AAI (with untrained animal)	Quiet space outside the regular classroom at school	Quantitative study with control group (typically developed children)	Treatment: M = 24, F = 9; Mean age = 9.4 (*SD* = 2.3; Aged 5.2–12.1) Control: M = 28, F = 38; Mean age = 9.0 (*SD* = 2.3; Aged 5.1–12.7)	99 (33/66)	Reduced general arousal (SCL) and the number of arousal peaks (SCRs) Reduced physiological arousal during peer interaction and induced positive emotions when animals were present
71	Ozyurt et al. (2020)	Turkey	Equine-assisted activity	Horse	AAI (with untrained animal)	Horse riding setting	Quantitative study with control group (no equine-assisted activity)	Treatment: M = 8, F = 4 Control: M = 9, F = 3 Aged 4 -12	24 (12/12)	Significant improvement in social communication and global functioning
72	Pan et al. (2019)	USA	Therapeutic horseback riding	Horse	AAI (with untrained animal)	Horse riding centre	Quantitative study with control group (no horse interaction barn activity)	Treatment: M = 6, F = 2; Mean age = 11.88 (*SD* = 2.45) Control: M = 7, F = 1; Mean age = 9.80 (*SD* = 2.82)	16 (8/8)	Significant improvement in hyperactivity, social awareness, significant improvements in irritability and social communication behaviors Significant reduction of stress levels as measured by salivary cortisol levels
73	Peters et al. (2020)	USA	Occupational therapy in an equine environment	Horse	AAI (with untrained animal)	Horse riding centre	Quantitative study	M = 5, F = 1 Aged 6–13	6	Significant improvement in occupational performance goals, social motivation and social communication
74	Peters et al., (2022a)	USA	Therapeutic horseback riding	Horse	AAI (with untrained animal)	Horse riding centre	Quantitative study with barn activity control group	Treatment: M = 37, F = 8; Mean age = 10.1 (*SD* = 2.8) Control M = 40, F = 4; Mean age = 10.4 (*SD* = 3)	89(45/44)	Significant improvement in self-regulation, social communication and number of new words spoken
75	Peters et al., (2022b)	USA	Occupational therapy in an equine environment	Horse	AAI (with untrained animal)	Horse riding centre	Quantitative study with waitlist control group	Treatment: M = 10, F = 2; Mean age = 8.68 (*SD* = 2.09) Control: M = 6, F = 3; Mean age = 9.45 (*SD* = 1.62)	21(12/9)	Significant improvement in social motivation and reduction in irritability
76	Petty et al. (2017)	USA	Therapeutic horseback riding	Horse	AAI (with untrained animal)	Horse riding centre	Quantitative study with barn activity control group (no-horse barn activity with no horse contact)	Treatment: M = 27, F = 4; Mean age = 10.95 (*SD* = 3.42) Control: M = 33, F = 3; Mean age = 10.01 (*SD* = 2.66)	67(31/36)	Significant improvement in the relationship with family pets, increase in caring manner towards family pets, increase in positive interactions with family pets
77	Prothmann et al. (2009)	Germany	Interaction with a therapy dog	Dog	AAI (with trained animal)	Carpeted video laboratory	Quantitative study (within group comparison)	M = 11, F = 3 Aged 6–14	14	Significant increase in social contact with the dog Significant decrease in socially isolated and self-stimulated behaviors
78	Salgueiroet al. (2012)	Portugal	Dolphin-assisted therapy	Dolphin	AAI (with trained animal)	Artificial lagoon	Quantitative study	M = 8, F = 2 Mean age = 6:9 (*SD* = 2:9)	10	Significant improvements in non-verbal communication, fine motor development and cognitive performance
79	Sams et al. (2006)	USA	Occupational therapy incorporating animals	Non-specific (dog, rabbit)	AAI (with untrained animal)	School	Quantitative study with control group (standard occupational therapy session)	Gender not mentioned Mean age = 9.6 (*SD* = 1.7; Aged 7–13)	22	Significantly greater use of language and social interaction
80	Silva et al. (2011)	Portugal	Interaction with a therapy dog	Dog	AAI (with trained animal)	Clinical setting	Quantitative study (within-subject design)	M = 1 Aged 12	1	Exhibited more frequent and longer durations of positive behaviors (e.g. smiling and positive physical contact) as well as less frequent and shorter durations of negative behaviors (aggressive manifestations)
81	Silva et al. (2018)	Portugal	Free play with a dog	Dog	AAI (with trained animal)	Home	Quantitative study (within-subject design)	M = 10 Aged 6–9	10	Larger proportions of committed compliance and lower proportions of passive non-compliance in the dog condition than in toy and robotic conditions Significantly more calmness and better latency to distress in the dog condition
82	Smyth and Slevin (2010)	Ireland	Ownership of an autism assistance dog	Dog	Pet ownership (with trained animal)	Home	Qualitative study	M = 8, F = 2 Aged 5–12	7	Being able to use more public areas, more family outings, enhanced communication, more social inclusion of child Improvement in morale, coping skills and peace of mind, increase in self-esteem, reduction in tantrums, depression and anxiety, no stress headaches
83	Solomon (2010)	USA	Animal-assisted therapy	Dog	AAI (with trained animal)	Home	Qualitative study	M = 4, F = 1 Aged 4–14	5	Being able to express the own meaning and intentions, make social initiation with unfamiliar children on the playground or in any setting More communication and participation in the family’s life
84	Souza-Santos et al. (2018)	Brazil	Equine-assisted activity	Horse	AAI (with trained animal)	Outdoor place	Quantitative study with control group (dance therapy, dance & equine-assisted therapy)	Each group: M = 12, F = 3 Each group: Mean age = 7 (*SD* = 1.09)	45 (15/15/15)	Reduction in the ASD symptoms and social participation
85	Steiner and Kertesz (2015)	Hungary	Therapeutic horseback riding	Horse	AAI (with untrained animal)	Open area	Quantitative study with control group (non-riding)	Treatment: M = 6, F = 7; Control: M = 6, F = 7 Aged 10–13	26 (13/13)	Improvement in communication skills (language numbers), usage of papers, self-care (washing wearing, traffic, eating), adaptive skills/socialization (housework, games)
86	Stevenson et al. (2015)	UK	Interaction with a dog	Dog	AAI (with untrained animal)	School dinner hall	Quantitative questionnaires & assessment, qualitative observations	M = 3 Aged 7–13	3	Increase in the levels of interaction, visual interest and meaningful vocalizations in the sessions with the dog Reduction in playing alone and sensory/repetitive behaviors, more focused and meaningfully engaged in sessions Generalized to classroom setting, being more engaged and interactive with their teacher
87	Tan and Simmonds (2018)	Australia	Equine-assisted intervention	Horse	AAI (with untrained animal)	Horse riding centre	Qualitative study	M = 1, F = 5 Aged 3–14	6	Formation of relationships with horses and with practitioners at sessions Learning of social skills with improvement in social motivation and quality of interaction in a positive social environment
88	Tepper et al. (2022)	Australia	Animal-assisted intervention	Dog	AAI (with trained animal)	Intervention service centre	Quantitative study	M = 9, F = 7 Mean age = 3.51 (*SD* = 0.5)	16	No significant improvement in social communication skills, executive functions and play type in any conditions of the therapy dog (being passive, active and absent from the session) With the presence of the therapy dog, children were more likely to remain stationary than engage in physical activities, which may indicate a global calming effect
89	Tseng (2023)	USA	Ownership of an autism assistance dog	Dog	Pet ownership (with trained animal)	Home	Quantitative study with repeated measure design	M = 9, F = 2 Mean age = 9.1 (*SD* = 1.5)	11	Improvement in social responsiveness (social recognition, social communication and social motivation) and behavioral problems (anxious/depressed condition, social problem and attention problem, as well as internalizing and externalizing problem) Reduction in chronic stress through an evaluation of a biological marker
90	van der Steen et al. (2019)	The Netherlands	Animal-assisted intervention	Horse	AAI (with untrained animal)	Rural wooded area	Quantitative and qualitative studies	F = 1 Aged 8	1	Improvement in social and communication skills, ability to deal with own body, emotion differentiation & emotion regulation, peer relationships & prosocial behaviors
91	Viau et al. (2010)	Canada	Interaction with a service dog	Dog	AAI (with trained animal)	Home	Semi-quantitative study with open-ended questionnaire	M = 37, F = 5 Mean age = 7.1 (*SD* = 3.1)	42	Decrease in the number of problematic behaviors reported by parents, frequency of self-stimulation episodes, repetitive behaviors and tantrums after the introduction of the dogs
92	Ward et al. (2013)	USA	Therapeutic horseback riding	Horse	AAI (with untrained animal)	Therapeutic horse riding centre	Quantitative study with single group quasi-experimental design	M = 15, F = 6 Mean age = 8.1	21	Significant increase in the social interaction, improvement in sensory processing and decrease in the severity of symptoms associated with ASD
93	Ward et al. (2017)	USA	Pet ownership	Non-specific (cat, dog, rodent, fish, reptile/ amphibian, rabbit, bird)	Pet ownership (with untrained animal)	Home	Quantitative study	M = 64, F = 9 Mean age = 13.91 (*SD* = 1.75)	73	Adolescents with greater social impairment, turning to pets for companionship was associated with higher friendship quality, but for those with less social impairment, turning to pets for companionship was associated with lower friendship quality Adolescents who took more responsibility for their pet exhibited fewer depressive symptoms
94	Wright et al. (2015a)	UK	Ownership of a dog	Dog	Pet ownership (with untrained animal)	Home	Quantitative study with control group (without a pet dog)	Treatment: M = 34, F = 8 Control: M = 21, F = 7 Mean age = 8.67 (*SD* = 3.34)	70 (42/28)	Anxiety scores in the dog-owning group reduced by a greater percentage, mostly in the domains of obsessive compulsive disorder, panic attack and agoraphobia, social phobia and separation anxiety
95	Wright et al. (2016)	UK	Pet ownership	Dog	Pet ownership (with untrained animal)	Home	Qualitative study	Dog owners: M = 16, F = 4 Non-dog owners: M = 14, F = 6 Mean age = 8.75 (*SD* = 3.47; Aged 3–15)	40	Dogs can provide companionship, enjoyment, fun and happiness to children, with increased confidence and social engagement with others
96	Zhao et al. (2021)	China	Therapeutic horseback riding	Horse	AAI (with untrained animal)	Equestrian training centre	Quantitative study with control group (routine activities)	Treatment: M = 21, F = 10; Mean age = 7.06 (*SD* = 1.5) Control: M = 23, F = 7; Mean age = 7.13 (*SD* = 1.36)	61 (31/30)	Significant improvement in social skills and social communication within the treatment group from pre-test to post-test Greater improvement in social interaction skills, responsibility and self-control over time compared with control group
97	Zoccante et al. (2021)	Italy	Equine-assisted activity	Horse	AAI (with untrained animal)	Horse valley (equestrian training centre)	Quantitative study	M = 13, F = 2 Aged 7–15	15	EAAT was associated with greater adaptive behavior (in terms of communication, daily living skills, socialization and motor skills), coordination and behavioral progressive improvement (in terms of social interaction, emotions-relation, behavior, gross motor skills and fine motor skills)

### Country of Origin

Among the 97 selected studies, 35 were conducted in Europe, while 32 were conducted in the US (Table 3). The remaining studies were conducted in Canada (*n* = 8), Asia (*n* = 6), and Australia (*n* = 10).

**Table 3:** Type of companion animal, setting and format of HAI in each country (N = 97)

		Countries					
	*N* (%)^a^	USA	Canada	Europe	Asia	Australia	Others^b^
Number of studies	97	32	8	35	6	10	6
Type of companion animal							
Dog	41 (42.7%)	9	6	13	4	6	3
Horse	34 (34.4%)	16	2	11	1	1	3
Guinea pig	7 (7.3%)	–-	–-	4	–-	3	–-
Cat	2 (2.1%)	2	–-	–-	–-	–-	–-
Dolphin	4 (4.2%)	–-	–-	3	1	–-	–-
Others^c^	9 (9.4%)	5	–-	4	–-	–-	–-
Type of setting							
Home	29 (30.2%)	12	5	9	–-	3	–-
Horse riding or training centre	29 (29.2%)	14	2	9	1	1	2
Clinical or treatment centre	11 (11.5%)	2	1	5	–-	2	1
School	11 (11.5%)	2	–-	2	3	3	1
Experimental setting or laboratory	3 (3.1%)	–-	–-	2	1	–-	–-
Outdoor spaces, farms or lagoon	8 (8.3%)	–-	–-	6	–-	–-	2
More than one setting	2 (2.1%)	1	–-	–-	1	–-	–-
Not mentioned	4 (4.2%)	1	–-	2	–-	1	–-
Format of HAI							
Pet ownership							
With trained animals	10 (10.4%)	2	4	2	–-	2	–-
With untrained animals	14 (14.6%)	9	–-	4	–-	1	–-
AAI							
With trained animals	29 (30.2%)	4	2	11	5	3	4
With untrained animals	44 (44.8%)	17	2	18	1	4	2

^a^Each percentage represents the number of studies in that category divided by the total number of studies (i.e. *N* = 97)

^b^Others include Brazil, Israel, Iran, and Jordan

^c^Others include non-specific animals or involving more than one companion animal in the study

### Types of Companion Animals and Settings

Within the selected studies, dogs were the most common type of companion animal involved (*n* = 41), followed by horses (*n* = 34) and guinea pigs (*n* = 7). Other animals included cats (*n* = 2) and dolphins (*n* = 4). Some studies involved unspecified companion animals or a mix of more than one type of animal (*n* = 9; Table 3).

As documented in the selected papers, HAI occurred in various settings. Most studies took place in home settings (*n* = 29) and horse riding or training centres (*n* = 29). Eleven studies were conducted in clinical and educational settings respectively, such as treatment centres and schools. Eight studies took place in outdoor spaces, farms, or lagoons. Three studies were conducted in experimental or laboratory settings. Two studies involved more than one setting, while the settings were not mentioned in the remaining four studies (Table 3).

### Formats of HAI and Types of Companion Animals Involved

Across the 97 selected studies (Table 3), two formats of HAI were identified: (1) pet ownership and (2) animal-assisted interventions. Animal-assisted intervention was the most common form of HAI (n = 73), comprising over 75% of all the selected studies. Among the AAI studies, 44 involved using companion animals with no specific training, while 29 involved trained service animals. Pet ownership was examined in 24 studies, with 14 involving untrained animals and the remaining ten involving trained animals.

The types of companion animals varied across the different HAI formats (Table 4). Dogs were primarily involved in studies on both pet ownership and AAI. All studies on pet ownership with trained animals utilized dogs as companion animals (*n* = 10). Among the 14 studies on pet ownership with untrained animals, the companion animals were more diverse, including dogs (*n* = 6), cats (*n* = 2), and unspecified or mixed types of animals (*n* = 6). Among the 29 AAI studies with trained animals, a majority involved dogs (*n* = 24), followed by horses (*n* = 3) and dolphins (*n* = 2). In the 44 AAI studies with untrained animals, horses were the most frequent companion animals (*n* = 31), followed by guinea pigs (*n* = 7).

**Table 4:** *Type of companion animal involved in different formats of HAI (N* = *97)*

	Format of HAI			
	Pet ownership		AAI	
Companion Animal	With trained animals	With untrained animals	With trained animals	With untrained animals
Dog	10	6	24	1
Horse	–-	–-	3	31
Guinea pig	–-	–-	–-	7
Cat	–-	2	–-	–-
Dolphin	–-	–-	2	2
Others^a^	–-	6	–-	3
Total	10	14	29	44

^a^Others include non-specific animals or involving more than one companion animal in the study

### Companion Animals, Settings, and Formats of HAI Across Countries

The studies on HAI revealed specific patterns in the types of companion animals, settings, and formats across different countries (Table 3). Around one-third of the 41 studies involving dogs were conducted in Europe (*n* = 13). Nearly half of the studies with horses were from the US (*n* = 16), followed by Europe (*n* = 11). Studies on guinea pigs came from Europe (*n* = 4) and Australia (*n* = 3). Both studies involving cats were carried out in the US. Three of the four studies on dolphins were from Europe, while one was from Asia. Studies with non-specific or multiple companion animals were mainly from the US (*n* = 5) and Europe (*n* = 4).

In terms of settings across countries (Table 3), over 40% of studies conducted at home (*n* = 12) were based in the US, while about half of the studies carried out at horse riding or training centres (*n* = 14) were located in the US. Around half of the studies in clinical or treatment centres were conducted in Europe (*n* = 5). Among the school-based studies (*n* = 11), three were conducted in Asia and three in Australia. Two out of the three experimental or laboratory studies were conducted in Europe, while one took place in Asia. Among the eight studies with outdoor or farm settings, six took place in Europe. Two studies involving multiple settings were conducted in the US and Asia, respectively, while the four studies without specified settings came from the US (*n* = 1), Europe (*n* = 2) and Australia (*n* = 1).

Regarding HAI formats across different countries (Table 3), 40% of the pet ownership studies with trained animals were conducted in Canada (*n* = 4), while over 60% of the pet ownership studies with untrained animals were conducted in the US (*n* = 9). Around 40% of the AAI studies were from Europe, with trained animals (*n* = 11) and untrained animals (*n* = 18). Among the six studies from Asia, all were related to AAI, with five studies involving trained animals and one study involving untrained animals. These results suggest variations in research interests and approaches to HAI across different geographical locations, focusing on pet ownership in the US and therapeutic applications involving companion or service animals in Europe and Asia.

## Discussion

This study aims to synthesize and refine current knowledge on HAI involving children with ASD across different countries and examine the potential cultural variations in HAI for better practice. The findings revealed that distinct patterns of HAI exist across countries, with cultural influences evident in the choice of companion animals, the format of HAI, and contextual constraints. Moreover, a suitable match between companion animals and children with ASD is crucial for the effectiveness of HAI. Hence, cultural factors and appropriate pairing of companion animals should be carefully considered to align with therapeutic goals to facilitate more adaptive HAI practices for children with ASD.

### Cultural Variations of HAI Across Countries

The results of this review have revealed interesting patterns regarding the formats and companion animals involved in HAI across different countries. These variations could be attributed to the cultural norms that shape attitudes towards animals.

The current review found that nearly half of the HAI studies conducted in home settings were from the US. It is believed that Western cultures tend to value companion animals more sentimentally, often anthropomorphizing them as family members who provide emotional support and love (Risley-Curtiss et al., 2006; Sheade & Chandler, 2014; Smith, 2019). This aligns with recent statistics showing that pet ownership is common in American households. According to the 2021–2022 APPA National Pet Owners Survey, around 70% of the US population owned at least one pet, with dogs being the most commonly owned companion animals. Specifically, it is a prevalent trend for American parents of children with ASD to explore complementary and alternative methods of treatment (CAM) (Volkmar et al., 2014). Among these treatments, animal-based intervention was found to be one of the most common CAM treatments, with one-quarter of children with ASD participating in AAI during childhood (Christon et al., 2010). According to Carlisle (2014), over 25% of dog-owning parents considered the benefits of dog ownership to their children with ASD to be a significant factor influencing their decision to own a dog. Hence, viewing pet ownership as a natural intervention for children with ASD likely explains the research interests in examining the daily impact of HAI on children with ASD in American household settings.

Moreover, nearly half of the HAI studies involving horses were conducted in the US. This reflects the long-standing tradition of therapeutic horseback riding as a recreational activity in the country, which helps horse riders develop skills in horseback riding and achieve both therapeutic and life goals (McDaniel Peters & Wood, 2017). Initially positioned for recreational purposes, it was adopted by occupational and speech therapists and subsequently embraced by various therapeutic professionals (Dismuke, 1984; Engel, 1984). Incorporating horses into recreational therapy is a common practice in the US, with certified therapeutic recreational specialists primarily serving individuals with neurodevelopmental disabilities such as autism (McKissock et al., 2023). Given the diverse therapeutic applications and popularity of human-horse interactions for children with ASD in the US, it is not surprising that a significant portion of relevant HAI research has been carried out in the country.

In this review, Europe was found to have the greatest number of HAI studies compared to other regions. The human–animal bond has long been considered an inherent connection with therapeutic power across European countries. Therapeutic support from animals for patients was first documented in a Belgian hospital in the eleventh century, and the involvement of companion animals has been widely used in different European healthcare institutions since the eighteenth century (Grandgeorge & Hausberger, 2011). The view of animals as sentient beings capable of experiencing emotions has further led to the enactment of various animal welfare and protection legislation in European countries like Austria, Germany, Switzerland, France, and Spain. These legal reforms have challenged the traditional view of animals as mere property, granting better protection for animals in civil codes (Cardoso et al., 2017). Hence, the focus on fostering a positive human–animal bond may have prompted further exploration of HAI through research studies, contributing to the phenomenon of a higher concentration of HAI studies conducted in Europe.

In recent years, green care has flourished across European countries, with AAI involving companion animals becoming a common type of green care intervention. Green care encompasses various therapeutic interventions that involve experiencing the natural environment and interacting with animals, such as care farming, healing gardens, eco-therapy, and AAI (Haubenhofer et al., 2010). For example, the United Kingdom has focused on horticultural therapy, while AAI with companion animals is preferred in countries like Finland, Norway, Germany, and Austria. In Germany, care farming has developed a strong link with animal husbandry, gardening, and the healthcare industry to promote physical and mental well-being through farming activities. The therapeutic context pertained to green care may explain the observation that most studies involving AAI were conducted in Europe, regardless of whether trained animals are involved.

Apart from therapeutic intentions, outdoor learning approaches have been widely adopted by many European countries, particularly in Scandinavia and the Nordic regions, where nature is considered a cultural symbol (Gullestad, 1992). Experiential education is closely tied to outdoor education in the UK and other European countries, which encompasses learning through outdoor activities, therapeutic interventions, and environmental education in natural settings (Änggård, 2010; Higgins, 2009). The emphasis on experience-based and sensory exploration in natural environments may account for the prevalence of HAI studies in outdoor spaces and natural settings in Europe. Research also suggests that dogs and horses are the most common companion animals involved in AAIs for children with ASD (Harris & Williams, 2017). While dogs are often trained as therapy animals for educational or clinical purposes, horses are widely integrated into outdoor therapeutic sessions for improving the social, behavioral, and motor skills of children with ASD. Thus, the unique therapeutic approach in Europe, which emphasizes AAI and outdoor settings, may elucidate why dogs and horses are the predominant companion animals in European HAI studies for autism interventions.

In contrast to the prevalence of HAI studies in the US and Europe, our results revealed that HAI studies were least documented in Asia. This is not surprising, as some Asian societies may be less comfortable with HAI due to certain cultural norms and demographic constraints. For example, China has a “one dog policy” that restricts the domestication of large dogs due to fears of rabies (Valiyamattam et al., 2018). In other Asian societies like Hong Kong, people are less likely to keep pets due to socioeconomic and environmental constraints, such as limited living space and the high cost of pet care (Chandler, 2022).

Although there is a growing interest in companion animals and concerns for animal welfare in Japan, the development of AAI still lags behind that of Western countries. For example, a society dedicated to therapeutic riding was established in Japan only in 2006 to support individuals with disabilities (Japan Therapeutic Riding Association, 2024). Moreover, there is a lack of research studies validating the effectiveness of equine-assisted activities and therapies for children with ASD in Japan (Kawamura et al., 2024), which may hinder the promotion of HAI in the region.

Despite the growth of therapy programs involving companion animals in Asian countries, cultural disapproval of companion animals and other practical constraints may continue to impede the development of AAI or HAI in these societies. The prevalent use of therapy animals in Asian countries may explain why most Asian studies focused primarily on AAI and service animal interactions rather than on pet ownership. Concerns and challenges related to pet ownership for children with ASD in Asian countries are underexplored and warrant examination in future studies.

### Association Between the Format of HAI and the Type of Companion Animals Involved

In this review, patterns have been observed that reveal associations between HAI formats and companion animals’ involvement. The functional roles supported by each animal species can be considered influential factors in determining the types of companion animals involved in the corresponding HAI. Optimal matching between companion animals and children with ASD is essential for achieving therapeutic goals and meeting mutual needs during interactions. Regardless of their training, companion animals serve significant functions that can be classified as either daily supportive or therapeutic.

First, the daily supportive roles of companion animals mainly involve providing emotional support and assisting with the daily functioning of children with ASD or their families. Some companion animals may not undergo specific training, yet they can still perform supportive functions for children with ASD. These animals are seen as sources of emotional attachment and facilitators of strong family bonds (Carlisle et al., 2023). Emotional attachment to small household animals, such as dogs and cats, is common in pet ownership. Many pet owners experience emotional support and a reduction in daily stress through the presence of companion animals, which primarily function as companions or sources of recreation, bringing joy and alleviating loneliness in daily life (Mueller, 2014).

For example, Wright et al. (2015a, 2015b) emphasized that pet dogs can serve as a common topic of interest and create shared family activities such as dog walking, thereby uniting the family and strengthening family bonds. In contrast, cats generally exhibit more independent and less intrusive behaviors than dogs, which may be preferable for families with children who have ASD (Carlisle et al., 2023). Children who view pets as siblings are often more willing to disclose their feelings to pets than to human siblings (Bures et al., 2021a; Power, 2008).

Some household pets are trained as therapy animals or service animals to enhance the well-being of individuals through HAI, especially for those with physical or mental disabilities. Among HAI studies focused on children with ASD, it has been revealed that most dogs involved are certified therapy dogs or designated autism assistance dogs that provide support for these children. For example, autism assistance dogs in home settings can offer daily support for families with children who have ASD, such as facilitating public outings and promoting family harmony.

In addition to daily support, the therapeutic role is another significant function performed by companion animals during interventions. Within AAI, the therapeutic goals for children with ASD may help determine the types of companion animals involved in the corresponding format of HAI. For example, interaction with dogs is often incorporated into interventions to enhance the social competence of children with ASD. Trained therapy dogs involved in AAI exhibit playful and responsive traits that enable them to fulfill the therapeutic role of speech elicitors among children with ASD during interventions (Fung & Leung, 2014). When children with ASD display emotional meltdowns or inappropriate social behaviors, service dogs may help divert their attention and provide social support (Smyth & Slevin, 2010). This may partly explain why all HAI studies related to dogs in this review were associated with service animal interaction in the formats of pet ownership or AAI.

Although large animals, such as horses, are rarely kept as household pets, their unique characteristics enable them to fulfill other therapeutic functions, such as interventions aimed at achieving physical and social outcomes. Specifically, horseback riding is used as a treatment tool in hippotherapy to accomplish functional goals, such as improving motor balance control and facilitating social engagement (Ajzenman et al., 2013). For instance, in equine-assisted programs, grooming horses can enable children to develop social skills, such as responsibility (Mueller, 2014). While horses in rehabilitation programs need to be well-trained to perform specific gaits and exercises for individuals with motor issues (De Santis et al., 2017), horses involved in equine-assisted psychotherapy in some therapeutic centres in Australia were found to lack proper training (Nelson et al., 2016). This may explain why, in some HAI studies, untrained horses could still be involved in AAI without service animal interaction to achieve specific social and emotional outcomes among children with ASD.

Guinea pigs, a common pocket animal, are often kept as household pets. Due to their calm temperament and small size, they can assist in classroom learning in educational settings (Krskova et al., 2010). Since classroom pets need to be small animals that are easy to handle in cages or aquariums, guinea pigs are often considered one of the best classroom pets to support learning for young children (O'Haire et al., 2013b). This review showed that over half of the HAI studies related to guinea pigs were conducted in schools, where they were incorporated into reading programs and classroom activities to enhance social-emotional functioning for children with ASD (Krskova et al., 2010; O'Haire et al., 2015). Like horses, untrained guinea pigs could be involved in less formal and unstructured animal-assisted activities. This contrasts with therapies assisted by dogs, which are typically conducted by educators or facilitators skilled in fostering positive HAI for children with ASD.

Dolphins have recently emerged as companion animals in AAI due to their intellectual competence and high responsiveness. They can be trained for dolphin-assisted therapy (DAT) to help therapists in enhancing the cognitive and social competence of children with mental or intellectual disabilities, especially those with ASD. Dolphins can elicit happiness from people due to their natural, spontaneous, and playful traits (MdYusof & Chia, 2012). However, interactions with dolphins are often constrained to waterside locations like lagoons or dolphinariums, making it more challenging to incorporate them into AAI compared to other companion animals. This may explain the scarcity of HAI studies on dolphin interaction in the field.

This study has explored the distinct patterns of HAI across different countries and highlighted potential cultural influences on the formats of HAI and the types of companion animals involved among children with ASD. To facilitate effective HAI for these children, it is essential to ensure that the choice of companion animals—considering factors such as size and temperament—is suitable for achieving the expected benefits and therapeutic objectives (Sams et al., 2006). The therapeutic effects of HAI can vary significantly based on individual differences among children with ASD. For instance, in conjunction with dog ownership, more regular contact with horses has been associated with increased adaptability to daily routines, suggesting that dog ownership and exposure to various animal species may create synergistic benefits for children with ASD and their families (Hall et al., 2016). Therefore, when introducing HAI to children with ASD for positive outcomes, their personalized educational programs should be carefully tailored to address skill development, therapeutic needs, degree of autonomy, and individual preferences (Hurtb et al., 1999; Philippe-Peyroutet & Grandgeorge, 2018). In addition, effective facilitators, including therapists and parents, play a crucial role in supporting and guiding children with ASD in positive HAI, such as teaching them how to approach and build relationships with companion animals (Lisk et al., 2023). Given the constraints in various settings, it is also important to consider cultural factors and the appropriate matching of companion animals when providing adaptive HAI practices for children with ASD.

### Limitations

Despite the comprehensive synthesis of cultural differences in HAI presented in this review, several limitations must be addressed. First, only articles with full English texts were selected, which excluded relevant HAI studies from Asian countries that were not published in English. Moreover, the lack of published papers on HAI in certain countries may simply suggest a deficiency in research within this field. It is plausible that HAI practices are extensively adopted in those countries despite the lack of research. This may also indicate a potential discrepancy between research output and the actual implementation of HAI across countries, which could be further explored in future studies.

Second, as this paper serves as an overview of HAI studies that examined the social and emotional benefits for children with ASD, the critical search terms were limited to those related to social and emotional outcomes. However, HAI also offers other developmental benefits for children with ASD, such as positive behavioral changes and improved motor skills (Borgi et al., 2016), which are worth examining to obtain a thorough understanding of HAI for therapeutic purposes.

In addition, potentially effective therapeutic components of HAI that support children with ASD were not examined in this review. For example, the calmness and responsiveness of companion animals may facilitate social interaction among children with ASD during HAI. It would be valuable to investigate in detail how the traits of each companion animal are associated with the benefits of interaction in various formats of HAI for children with ASD.

Lastly, investigating the underlying mechanisms of HAI and how they are influenced by cultural contexts can provide a more concrete theoretical basis for understanding the cultural variations in HAI for children with ASD. Comparative studies of HAI between Eastern and Western countries could yield more rigorous validation.

## Conclusion

This study is one of the first to synthesize the current phenomenon of HAI for children with ASD and discuss its potential cultural variations across countries. The findings of this review reveal distinct patterns of HAI that may vary culturally based on societal norms, intervention approaches, and the corresponding constraints of different settings. Given this cultural diversity and its possible influence on HAI, parents, educators, and therapists should be aware of the cultural background and the flexibility of intervention approaches when facilitating HAI (Chandler, 2012). This review highlights the need to promote a more adaptive HAI tailored to the therapeutic needs of children with ASD while considering the possible influence of diverse cultural contexts and practical constraints. In light of cultural diversity, this review further serves as a reference for fostering a localized framework of HAI for children with ASD in each country. Future studies could further explore other normative factors, such as children with ASD’s attitudes toward companion animals, preferences, and biases about HAI. This would contribute additional conceptual knowledge to the current literature regarding the multicultural considerations of HAI for children with ASD in different countries.

## Data Availability

The authors confirm that the data supporting the findings of this study are available upon reasonable request.
